# Novel method to classify hemodynamic response obtained using multi-channel fNIRS measurements into two groups: exploring the combinations of channels

**DOI:** 10.3389/fnhum.2014.00480

**Published:** 2014-07-02

**Authors:** Hiroko Ichikawa, Jun Kitazono, Kenji Nagata, Akira Manda, Keiichi Shimamura, Ryoichi Sakuta, Masato Okada, Masami K. Yamaguchi, So Kanazawa, Ryusuke Kakigi

**Affiliations:** ^1^Department of Psychology, Chuo UniversityTokyo, Japan; ^2^Research and Development Initiative, Chuo UniversityTokyo, Japan; ^3^Japan Society for the Promotion of SciencesTokyo, Japan; ^4^Department of Complexity Science and Engineering, The University of TokyoKashiwa, Japan; ^5^Department of Pediatrics, Dokkyo Medical University Koshigaya HospitalKoshigaya, Japan; ^6^Center for Child Development and Psychosomatic Medicine, Dokkyo Medical University Koshigaya HospitalKoshigaya, Japan; ^7^RIKEN Brain Science InstituteWako, Japan; ^8^Department of Psychology, Japan Women’s UniversityKawasaki, Japan; ^9^Department of Integrative Physiology, National Institute for Physiological SciencesOkazaki, Japan

**Keywords:** hemodynamic data, near-infrared spectroscopy (NIRS), support vector machine (SVM), sparse modeling, attention-deficit/hyperactivity disorder (ADHD), autism spectrum disorders (ASD)

## Abstract

Near-infrared spectroscopy (NIRS) in psychiatric studies has widely demonstrated that cerebral hemodynamics differs among psychiatric patients. Recently we found that children with attention-deficit/hyperactivity disorder (ADHD) and children with autism spectrum disorders (ASD) showed different hemodynamic responses to their own mother’s face. Based on this finding, we may be able to classify the hemodynamic data into two those groups and predict to which diagnostic group an unknown participant belongs. In the present study, we proposed a novel statistical method for classifying the hemodynamic data of these two groups. By applying a support vector machine (SVM), we searched the combination of measurement channels at which the hemodynamic response differed between the ADHD and the ASD children. The SVM found the optimal subset of channels in each data set and successfully classified the ADHD data from the ASD data. For the 24-dimensional hemodynamic data, two optimal subsets classified the hemodynamic data with 84% classification accuracy, while the subset contained all 24 channels classified with 62% classification accuracy. These results indicate the potential application of our novel method for classifying the hemodynamic data into two groups and revealing the combinations of channels that efficiently differentiate the two groups.

## Introduction

Near-infrared spectroscopy (NIRS) has been utilized to measure brain activity in humans (for review, Ferrari and Quaresima, 2012). Because NIRS is non-invasive and requires less stabilization of participants than other neuroimaging techniques, NIRS is highly suitable for studies with infants, children (for review, Lloyd-Fox et al., 2010; Gervain et al., 2011) and patients with psychiatric symptoms or disorders such as schizophrenia, depression, anxiety disorder, and attention-deficit/hyperactivity disorder (ADHD) (for review, Fukuda, 2009; Ernst et al., 2012). ADHD is characterized by major symptoms of hyperactivity, impulsivity and inattention (American Psychiatric Association, 2000). Children with ADHD (ADHD children) show atypical hemodynamic response in the prefrontal region associated with attention and working memory deficits (Weber et al., 2005; Negoro et al., 2010; Monden et al., 2012, but see also Schecklmann et al., 2010).

The neural response of ADHD children in face processing is different from that of typically developing children (TD children). Tye et al. (2013) used ERP techniques to examine the face-inversion effect and gaze processing in ADHD children, children with ASD (ASD children), children with comorbid ASD+ADHD, and TD children. They found that children with ADHD (ADHD/ADHD+ASD) showed atypical response that reflect early attentional stage of face processing, while children with ASD (ASD/ASD+ADHD) showed atypical response in gaze processing and atypical neural specialization, which are likely to be more relevant to the characteristic social deficits of autism. As far as we know, Tye et al. (2013) was the first study that found the basic face processing in ADHD children different from that of ASD children and no previous studies has yet investigated familiar face processing in children with ADHD. Dawson et al. (2002) demonstrated that ASD children showed no differential ERPs when viewing their mother’s face and viewing an unfamiliar female face, while TD children did. Although the hemodynamic response to one’s mothers’ face has not been tested with ADHD children, we may suppose that an atypical response might also be observed in ADHD children.

Recently our group found that boys with ADHD showed a different hemodynamic response to their own mother’s face than typically developing boys (Shimamura et al., 2012, under review). Thorell et al. (2012) tested non-clinical 8.5 year-old children and reported that attachment disorganization and executive functioning were independently related to ADHD symptoms. Furthermore, Carlsson et al. (2008) investigated the endogenous and exogenous factors that predict inattentiveness and hyperactivity in middle childhood and demonstrated that the quality of the caregiving more powerfully predicted inattention and hyperactivity than did early biological or temperamental factors. The quality of the relationship between the child and the caregiver might increase or reduce the amount of communication between the two, even in TD children, which may possibly affect the development of the children’s neural basis for processing his/her mother’s face. (Incidentally, all adults participated as “caregivers” in Shimura et al.’s study were the mother’s of the children.)

Furthemore, we found that boys with ADHD showed a different hemodynamic response to their own mother’s face than boys with ASD (Shimamura et al., 2012, under review). Although ASD is characterized by difficulties with social interaction, restricted interests, and repetitive behaviors (American Psychiatric Association, 2000), there is a large overlap of symptoms in ADHD and ASD patients such as hyperactivity, restlessness, and impairments in social cognitive abilities (Yerys et al., 2009; Taurines et al., 2012; van der Meer et al., 2012). It is often difficult in actual clinical practice to distinguish between these patients due to their overlapping symptoms (Yoshida and Uchiyama, 2004). Using fNIRS, we presented boys of both groups with images of their mother’s face and measured their cerebral hemodynamics in the bilateral temporal area. Only children with ADHD showed a significantly greater concentration of oxy-hemoglobin (oxy-Hb) in the bilateral temporal area than the pre-task baseline; children with ASD showed a decrease of oxy-Hb concentration in the left temporal area.

These findings suggest the possibility of distinguishing the cerebral hemodynamic data of the ADHD participants from those of the ASD participants and the possibility of classifying the data into two separate diagnostic groups. With such classification, we might be able to predict to which diagnostic group an unknown participant belongs by analyzing his/her hemodynamic data.

To classify the hemodynamic data into two groups, a promising method involves the Support Vector Machine (SVM; Vapnik, 1982). The SVM is a multivariate method for binary classification and has recently been introduced to NIRS studies aimed at the development of BCI (Sitaram et al., 2007; Cui et al., 2010).

When using multivariate pattern analysis methods such as SVM, we can improve classification accuracy by selecting informative variables and eliminating uninformative ones (Weston et al., 2001; Guyon and Elisseeff, 2003; Liu and Yu, 2005). This process is called feature selection or variable selection and has been increasingly applied in recent BCI studies (Yamashita et al., 2008; Gottemukkula and Derakhshani, 2011). A number of feature selection methods have been proposed in past studies (Guyon and Elisseeff, 2003; Liu and Yu, 2005). These methods, however, do not necessarily select the subset of variables that gives the best classification accuracy because these methods are intended to be applied to high-dimensional data such as genetic data, and are designed to decrease computation time by using approximate treatment. Here, without approximate treatment, we exhaustively evaluated classification accuracy using all 2^24^ − 1 = 16,777,215 subsets of channels to find the best one, using 5-fold cross validation.

In this study, we applied SVM to real hemodynamic data obtained in Shimamura et al. (2012, under review) and tried to classify the data into two groups: ADHD participants and ASD participants. We exhaustively searched the optimal subset among all subsets of channels and evaluated the classification accuracy using 5-fold cross validation. To compare the effectiveness of SVM with a standard method, we applied two feature selection methods: Lasso (Tibshirani, 1996) and sparse logistic regression (SLR) (Yamashita et al., 2008), and a channel-wise *t*-test, which is one of the most popular statistical analysis to find an activated channel in NIRS measurement.

## Method and analysis

### Participants

The participants in this study were nine boys with ADHD (three ADHD-only and six with comorbid ADHD+ASD; mean age = 9y9m, *SD* = 1y7m) and eight boys with ASD (two ASD-only and six with comorbid of ASD+ADHD; mean age = 9y9m, *SD* = 1y4m). Six ADHD boys received methylphenidate, and one ADHD boy received atomoxetine. The mean score of ADHD-Rating Scale was 34.2 (range = 11–52; *SD* = 13.9) in the ADHD group, 26.6 (range = 16–38; *SD* = 7.6) in ASD group. The two-tailed two-sample *t*-test did not show significant difference in their ADHD-RS scores [*t*_(15)_ = 1.375, *ns*]. These two diagnostic groups were not different in their age or sex. All diagnoses were based on the DSM-IV-TR (American Psychiatric Association, 2000) and were made by a pediatric neurologist (Ryoichi Sakuta).

### Protocol

During the measurement, participants observed the visual stimuli presented on the monitor. A single trial was comprised of a baseline period and test period. Each trial started with a baseline period during which the participants fixated on a black dot displayed on the monitor at the rate of 1 Hz. The duration of the baseline period was at least 20 s. Following this, a test period began. During the test period, the image of the child’s mother’s face or that of an unknown female face was presented. Either the mothers’ face or the unknown female face appeared successively 10 times at the rate of 1 Hz. The duration of test trial was 10 s. The mothers’ face and the unknown face were presented alternatively. Each participant performed five trials. In this study, we analyzed the hemodynamic data only from when the boys were passively looking at their mother’s face.

The study was approved by the Ethical Committee of the Dokkyo Medical University Koshigaya Hospital and by the Ethical Committee of Chuo University. Written informed consent was obtained from the participants and their parent. The experiments were conducted according to the Declaration of Helsinki.

### Functional NIRS recording

We used a Hitachi ETG-4000 system (Hitachi Medical, Chiba, Japan), which can record from 24 channels simultaneously, with 12 channels for the right temporal area and 12 for the left. This instrument generates two wavelengths of NIR (695 and 830 nm) and measures the time courses of the levels of oxyhemoglobin (oxy-Hb), deoxyhemoglobin (deoxy-Hb), and their sum (total hemoglobin: total-Hb) with 0.1 s time resolution. Based on the previous study, which showed that the oxy-Hb change reflects the task-related neural activity more reliably than deoxy-Hb or total-Hb (Strangman et al., 2003; Cui et al., 2011), we analyzed only the oxy-Hb concentration.

**Figure 1:**
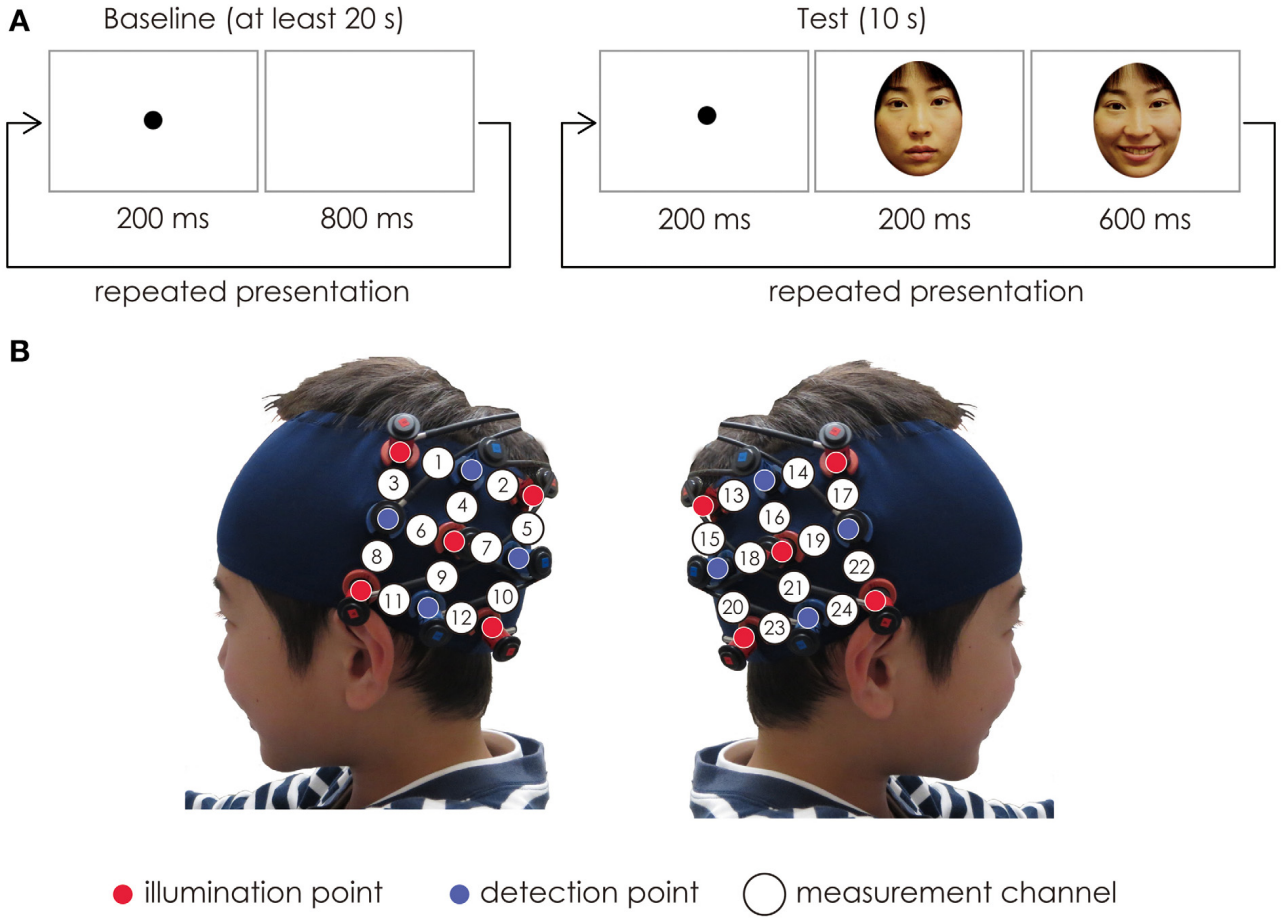
**(A)** The stimuli sequence. In each trial, the baseline period consisted of the black dot, and its duration was at least 20 s. The test period consisted of a happy expression of the mother’s face. The duration of the test period was fixed for 10 s. The baseline period and test period were presented alternatively. **(B)** Location of the probe and the measurement channels. The fibers were placed on the left and right temporal areas centering at the T5 and T6 of the International 10–20 system. The distance between the fibers was set at 3 cm.

The probes were set on the child’s scalp at the bilateral temporal area centered at T5 and T6 according to the International 10–20 system (Jasper, 1958) (Figure 1). When the probes were positioned, the experimenter checked to see if the fibers were touching the child’s scalp correctly. The Hitachi ETG-4000 system automatically detects whether the contact can adequately measure the emerging photons for each channel. All the trials were rejected from the analysis if adequate contact between the fibers and the child’s scalp couldn’t be achieved because of hair interference.

### Preprocessing of data

The raw data on oxy-Hb concentrations from each channel were digitally band-pass-filtered at 0.02–1.0 Hz to remove any noise due to heartbeat pulsations and any longitudinal signal drift.

Although the raw NIRS data were originally relative values, and could not be compared directly across subjects or channels, the normalized data such as the Z-score could be averaged regardless of the unit (Schroeter et al., 2003; Shimada and Hiraki, 2006). This calculation of the Z-score is a reliable analysis for changes in concentration in the children’s brains since the analysis is independent of the differential path length factor (DPF).

In order to show the relative change of oxy-Hb concentration during the task period, we standardized the raw data into Z-scores based on the baseline period ahead of the task period. We calculated the Z-scores of oxy-Hb in a time series of 0.1 s time resolutions from 3 s before the test period onset to the test period offset (Ichikawa et al., 2010; Kobayashi et al., 2011, 2012). The Z-score at each time point can indicate the deviation of hemodynamic response during the presentation of faces from the “baseline.” The “baseline” for calculating the Z-score was a period of 3 s immediately before the beginning of the each test period, which reflects the activation during the observation of the blank and fixation points. The Z-scores were calculated using the following formula:

(1) $$d=\left(x_{test}-m_{baseline}\right)/s$$

*x_test_* represents the raw data [mM mm] at each time point during the test period and *m_baseline_* represents the mean of the raw data during the baseline period. *s* represents the *SD* of the raw data during the baseline period.

Consistent with a previous study (Boynton et al., 1996) and our previous studies using NIRS (Ichikawa et al., 2010; Nakato et al., 2011), we found that a response peak lags a few seconds behind stimulus onset. Therefore, we performed the following analyses against the mean Z-scores from 3 to10 s after the face stimulus onset.

The Z-scores were calculated separately for each trial. In this study, we eliminated the trials from further analysis if all of 24 channels were not completely recorded due to hair interference or motion artifact. Finally, the number of data we obtained was 50: 25 data from 6 participants of the ADHD group and 25 data from 8 participants of the ASD group. The mean number of valid trials was 4.3 (*SD* = 2.7) per child with ADHD and 3.1 (*SD* = 1.3) per child with ASD.

### Support vector machine (SVM)

We trained the SVM to discriminate data by considering to which diagnosis group the owner of the data belongs. We used the mean Z-scores of hemodynamic activities and the diagnosis group of participants as inputs and outputs of the SVM, respectively.

SVMs are state-of-the-art models for classification with a high generalization capability (Vapnik, 1982; Bennett and Mangasarian, 1992; Cortes and Vapnik, 1995). Given input data, an SVM classifies them into two classes. An SVM learns the relationship between the input data and their classes from the training samples. It also predicts the class of unknown data.

(2) $${\left\{\left(x_{i},t_{i}\right)|x_{i}\in {\mathbb{R}}^{D},t_{i}\in \left\{+1,-1\right\}\right\}}_{i=1}^{N},$$

where *x_i_* is a D-dimensional feature vector, and *t_i_* is a class label of *x_i_*. In this study, the mean Z-scores were calculated for 24 channels (or 12 for hemispheric analysis) for each trial, and were used as a 24-dimensional (or 12-dimensional) feature vector. We had 50 valid trial data sets and 50 feature vectors input to the SVM. We related ADHD participants to *t_i_* = +1 and ASD participants to *t_i_* = −1.

Given this data set, an SVM finds a hyperplane in the feature vector space that separates the samples into two groups. The obtained hyperplane is called a decision boundary. New samples are then classified according to which side of the boundary they belong. Classifying samples using a decision boundary is a common practice for other linear classifiers such as logistic regression. What characterizes SVMs is margin maximizing. The margin is the distance between the decision boundary and the closest sample to the decision boundary. By maximizing the margin, SVMs are capable of accurately predicting the classes of new samples. The decision boundary is expressed as a linear equation as follows:

(3) $$y\text{​}\left(x\right)=w^{T}x+b=0$$

Here *w* is a weight vector. We want to find w and b that satisfy *y*(*x_i_*) > 0 for *t_i_* = 1 and *y*(*x_i_*) < 0 for *t_i_* = −1, that is, *ty*(*x*) > 0 for all samples (Vapnik, 1982). The larger value of *w* indicates that the more corresponding *x* contributes to classify data into two groups. A positive sign of *w* indicates that the larger value of a corresponding *x* raises the possibility of *t_i_* = 1, while a negative sign of *w* indicates that the larger value of a corresponding *x* raises the possibility of *t_i_* = −1.

### Cross validation

Cross validation (CV) is a method for evaluating how the ability of a learning machine such as an SVM is generalized to the unknown data that are not used in the training (Kohavi, 1995). In the CV, the data set is divided into two parts. One part is used for the training of the machine, and the other part is used for testing the machine’s ability. This training and testing procedure is repeated using different partitioning. The CV is effective when the number of the data is limited.

We explain the *K*-fold CV for the SVM below. First, we divide the data set into *K* parts *C*_1_,…, *C_K_*. For each *k* = 1,…, *K*, we train the SVM using the data other than the *k*-th part *C_k_*. We denote by *y\_k_* (*x*) the decision boundary. Then, by using this boundary *y\_k_* (*x*), we predict the classes of the data in *C_k_*, and compare them against the true class labels *t*. We repeat this procedure for every *k* = 1,…, *K*, and calculate the following cross validation error (CVE):

(4) $$\text{CVE=}\frac{1}{N}\sum_{k=1}^{K}\sum_{i\in C_{k}}L\text{​}\left(t_{i},y\backslash_{k}\left(x_{i}\right)\right),$$

(5) $$L\text{​}\left(t,y(x)\right)=\;\{\begin{array}{c} 1\;(ty\left(x\right)>0)\\ 0\;(ty\left(x\right)<0) \end{array}$$

*L*(*t, y*(*x*)) indicates whether the prediction is correct or not. When the prediction is correct, *L*(*t, y*(*x*)) = 0, and when the prediction is incorrect, *L*(*t, y*(*x*)) = 1. CVE represents the ratio of the number of incorrectly predicted data to the total number of data. If the CVE is small, the generalization capability of the SVM is high. The classification accuracy is defined using the following formula:

(6) (1−CVE)×100

We used a 5-fold CV. In this study, we used three data sets; (1) *D* = 12 (12 channels placed on the right temporal area), (2) *D* = 12 (12 channels placed on the left temporal area), and (3) *D* = 24 (24 channels placed on the bilateral temporal areas). The above sets of (1) and (2) were subsets of (3).

We selected a subset *A* of *D* features and set *x_i_A__*: = (*x_i_d__*)_*d*∈A_ ∈ ℝ^|*A*|^. We then applied the 5-fold CV to the data set {(*x_i_S__, t_i_*)}*^N^_i_*_= 1_ and calculated the CVE. We carried out this process for all (2^*D*^ − 1) subsets.

Our aim was to find the optimal subsets that classify the data into two groups most correctly. In other words, we wanted to find the combination of NIRS measurement channels that distinctively respond to the experimental stimuli depending on which diagnostic groups the data belong.

## Results

We used the mean Z-scores of hemodynamic response obtained from each channel and the diagnostic group of participants as inputs and outputs for the SVM, respectively. We trained the SVM to find the optimal subset in the data set of channels in order to classify the data into two diagnostic groups. For each subset, we evaluated its classification accuracy using 5-fold CV.

We conducted the exhaustive search of three data sets of Z-scores of hemodynamic data: (1) the 12-dimension dataset obtained from the right 12 channels, (2) the 12-dimension dataset obtained from the left 12 channels, and (3) the 24-dimension dataset obtained from the bilateral 24 channels. For the 12-dimension datasets [(1) and (2)], SVM training and calculation of the CVE was repeated with 2^12^ − 1 = 4095 subsets. For the 24-dimension dataset (3), SVM training and calculation of the CVE was repeated with 2^24^ − 1 = 16,777,215 subsets.

### Results of SVM classification applied on the data obtained from the right hemisphere

**Figure 2:**
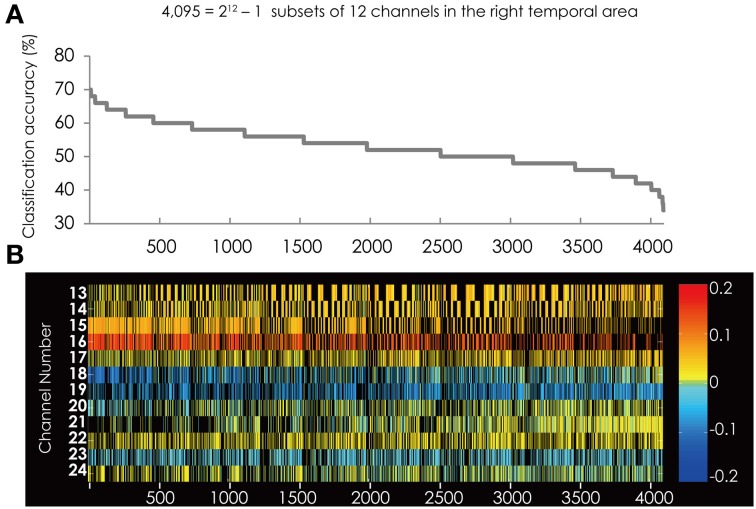
**(A)** The classification accuracy corresponding with 4095 subsets consisted from the channels in the right hemisphere. Horizontal axis represents the rank-ordered subsets. Vertical axis indicates the classification accuracy. The highest accuracy is represented on the left side. **(B)** The channel numbers in the subsets represented in **(A)**. Horizontal and vertical axes represent the rank-ordered feature subsets and the channel numbers, respectively. Colored cells indicate that the channel was in the subset and black cells indicate that the channel was not in the subset. The color of each cell indicates a value of weight vector corresponding to the Z-score of each channel. Red/yellow spectrum indicates positive value and blue spectrum indicates negative value.

We found nine subsets to classify the data more correctly into two groups among the 4095 subsets. The measurement channels which comprised those subsets are listed in Table 1. The best classification accuracy for these subsets was 70%. Figure 2A shows the classification accuracy for all 4095 subsets.

**Table 1:** **The measurement channels those comprised the subsets with best classification accuracy^a^**.

**Right**	**Left**	**Bilateral**
**(70%)**	**(74%)**	**(84%)**
15, 16, 18, 24	1, 6, 7, 9, 11	1, 3, 4, 5, 6, 8, 9, 10, 13, 14, 18, 20
15, 16, 17, 18, 24		1, 3, 4, 5, 6, 8, 9, 13, 14, 15, 18, 20, 22, 23
13, 15, 16, 17, 18, 19		
15, 16, 18, 20, 22, 24		
15, 16, 18, 20, 23, 24		
14, 15, 16, 17, 18, 20, 24		
15, 16, 17, 18, 20, 23, 24		
13, 15, 16, 17, 18, 20, 23, 24		
14, 15, 16, 17, 18, 20, 23, 24		

a *The numbers indicated the channel numbers. In the right hemisphere, the number of the best subset was nine. The percentage represented in the parenthesis indicating the best classification accuracy for each data set*.

Figure 2B shows the feature subsets corresponding to the classification accuracy represented in Figure 2A. Figure 2B shows that Ch. 15, 16, and 18 repeatedly appear in the subsets represented on the left side, which have relatively higher classification accuracy. These channels are filled with darker color, indicating these channels have a stronger coefficient than other channels. Furthermore, the colors of these three channels were consistent among all the subsets, respectively. This indicates that their weight vectors have the same sign commonly in all the subsets.

Moreover, Figure 2B shows that the red colored cells and blue colored cells are separated. The lower-numbered channels (Ch. 13–17) are constantly red and the higher-numbered channels (Ch. 18, 19, and 23) are constantly blue. This suggests that in the right hemisphere the brain area contributing to classification are separated. On the other hand, other channels were constantly filled with yellowish color. These channels do not have strong weight vectors, thus, they do not contribute to the classification in all of the 4095 subsets.

**Figure 3:**
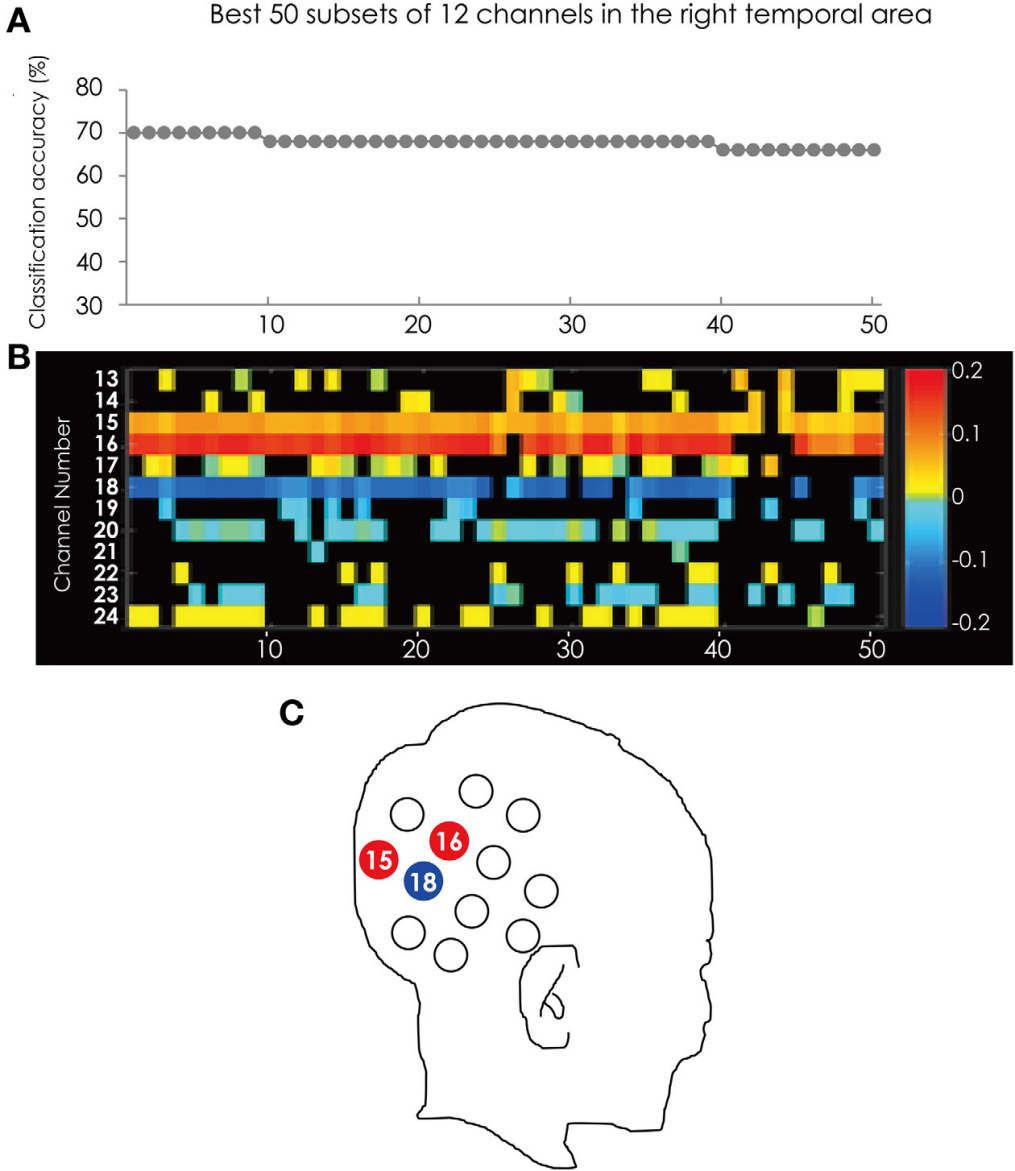
**(A)** The classification accuracy corresponding with the best 50 subsets consisted from the channels in the right hemisphere. **(B)** The channel numbers in the subsets represented in **(A)**. **(C)** The position of the channels referred in the text.

Figure 3 shows the classification accuracy of the best 50 subsets (Figure 3A) and the corresponding feature subsets (Figure 3B). Each cell indicates the weight vector of each channel in the feature subset. Ch. 15, 16, and 18 appear in most of subsets. Ch. 15 was in 49 of the 50 subsets. Ch. 16 was in 45 subsets and Ch. 18 was in 40 subsets, respectively. These three channels were more often used in the best 50 subsets and more effectively contributed to the classification. The positions of these channels are illustrated in Figure 3C.

The color of the cells corresponding to Ch. 15 and 16 are red, but Ch. 18 is blue. This tendency is consistent through the best 50 subsets. This result indicated that the greater hemodynamic response in Ch. 15 and 16 more often occurred in ADHD participants than in ASD participants, and those at Ch. 18 were more often found in ASD participants than in ADHD participants.

### Results of SVM classification applied on the data from the left hemisphere

**Figure 4:**
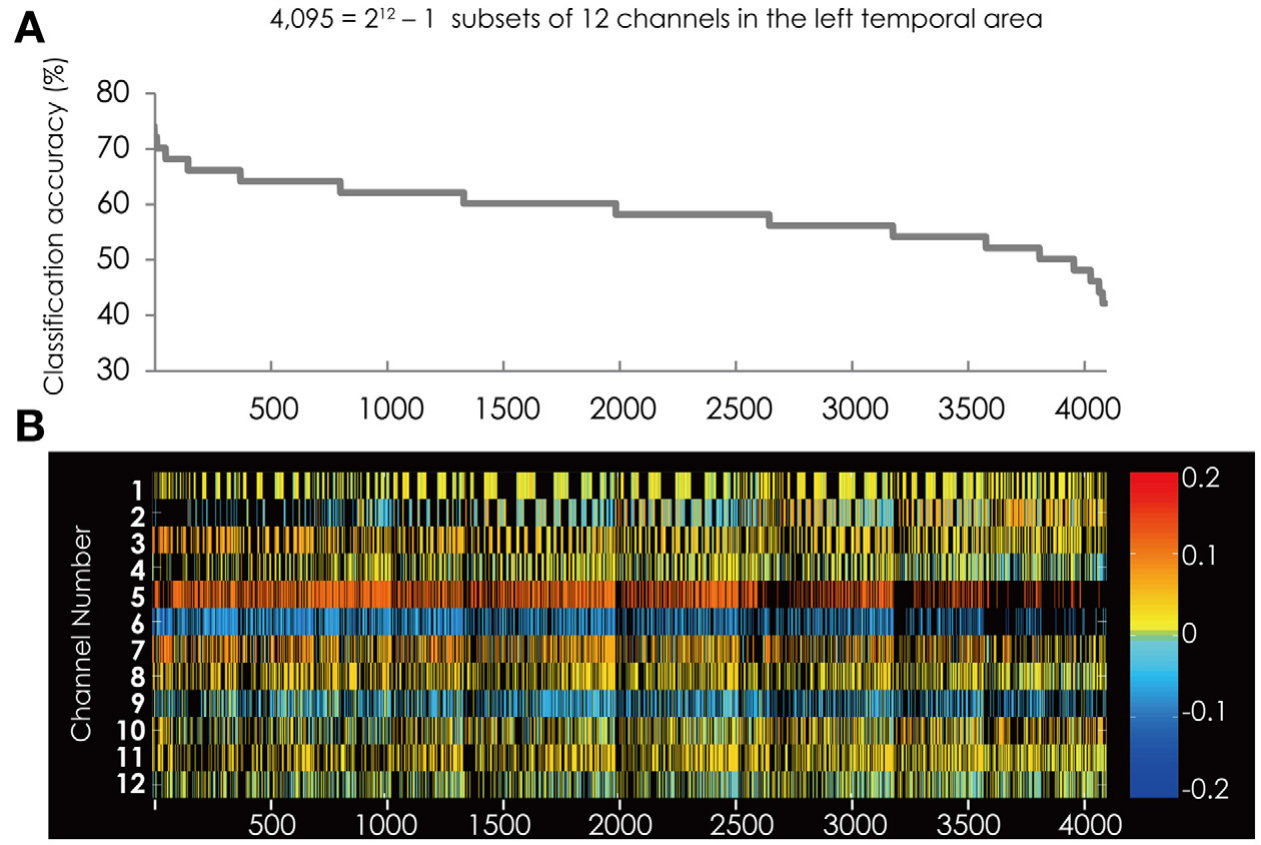
**(A)** The classification accuracy corresponding with 4095 subsets consisted from the channels in the left hemisphere. **(B)** The channel numbers in the subsets represented in **(A)**.

The best subset for classifying the data into two groups contained the five channels listed in Table 1. This subset had the best classification accuracy of 74%. Figure 4 shows the classification accuracy for all 4095 subsets (Figure 4A) and the corresponding feature subsets (Figure 4B). Ch. 5 and 6 constantly appear in the subsets with relative higher classification accuracy and had greater weight value than the other channels. Furthermore, the color of their cells is consistent through all the subsets. This indicates the sign of the weight vector is common in all the subsets.

Compared with the result from the right hemisphere (Figure 3B), the red colored cells and blue colored cell are scattered among the channels. This suggests that in the left hemisphere the brain areas contributing to classification are not clearly separated.

**Figure 5:**
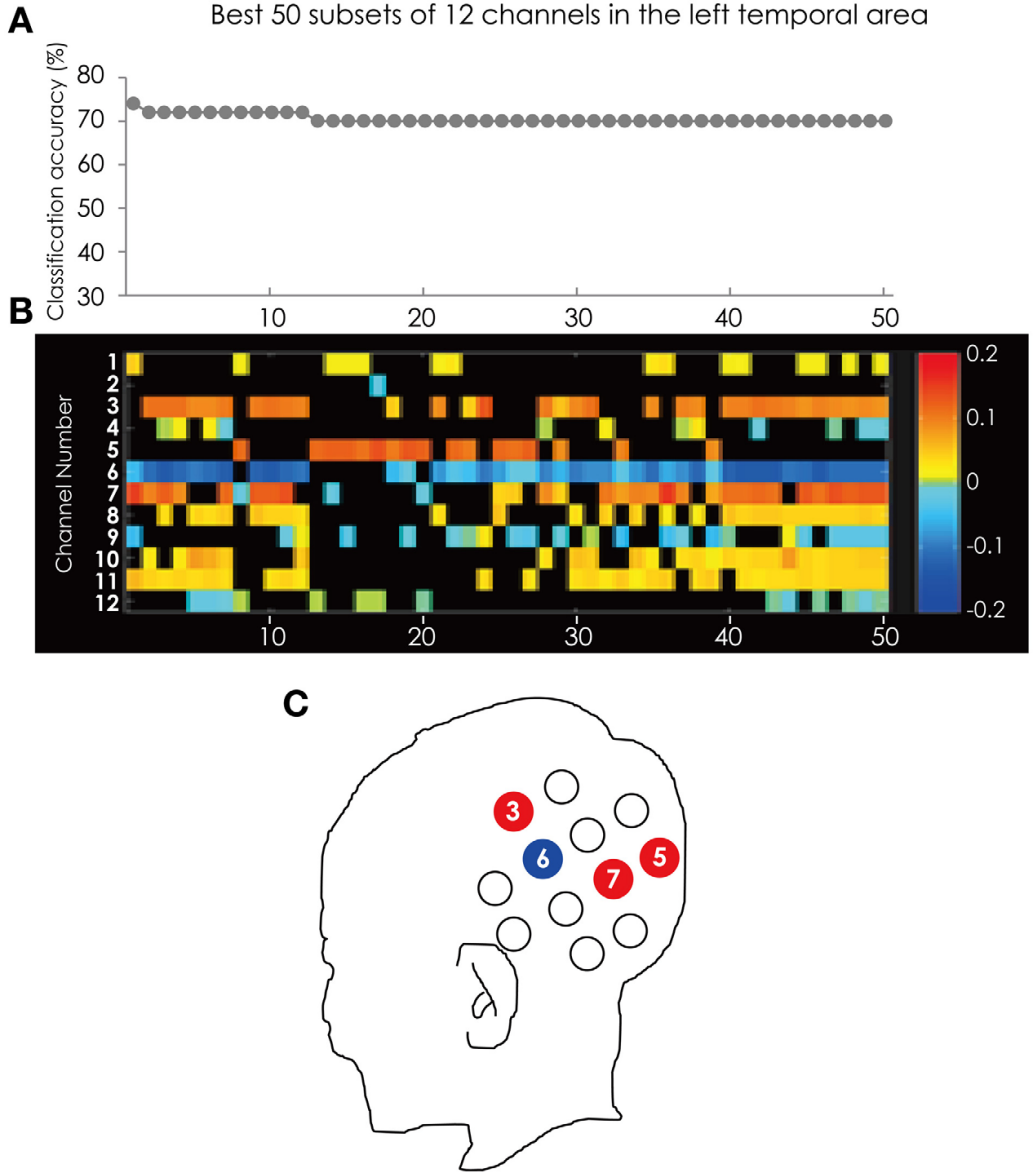
**(A)** The classification accuracy corresponding with best 50 subsets consisted from the channels in the left hemisphere. **(B)** The channel numbers in the subsets represented in **(A)**. **(C)** The position of the channels referred to in the text.

Figure 5 shows the classification accuracy for all 4095 subsets (Figure 5A) and the corresponding feature subsets (Figure 5B). Ch. 6 was most often used in the best 50 subsets. The channel was in 43 of the 50 subsets. Following Ch. 6, 3, and 7 were in 32 subsets. It is worth mentioning that Ch. 5 and 6 appear alternately in the top 17 subsets. The position of these channels is illustrated in Figure 5C.

Ch. 3, 5, and 7 are red and only Ch. 6 is blue. This tendency is consistent through the best 50 subsets. This result indicated that at Ch. 3, 5, and 7 ADHD participants showed greater hemodynamic response than ASD participants, and only at Ch. 6 did ASD participants show greater hemodynamic response than ADHD participants.

### Results of SVM classification applied on the data from bilateral hemispheres

**Figure 6:**
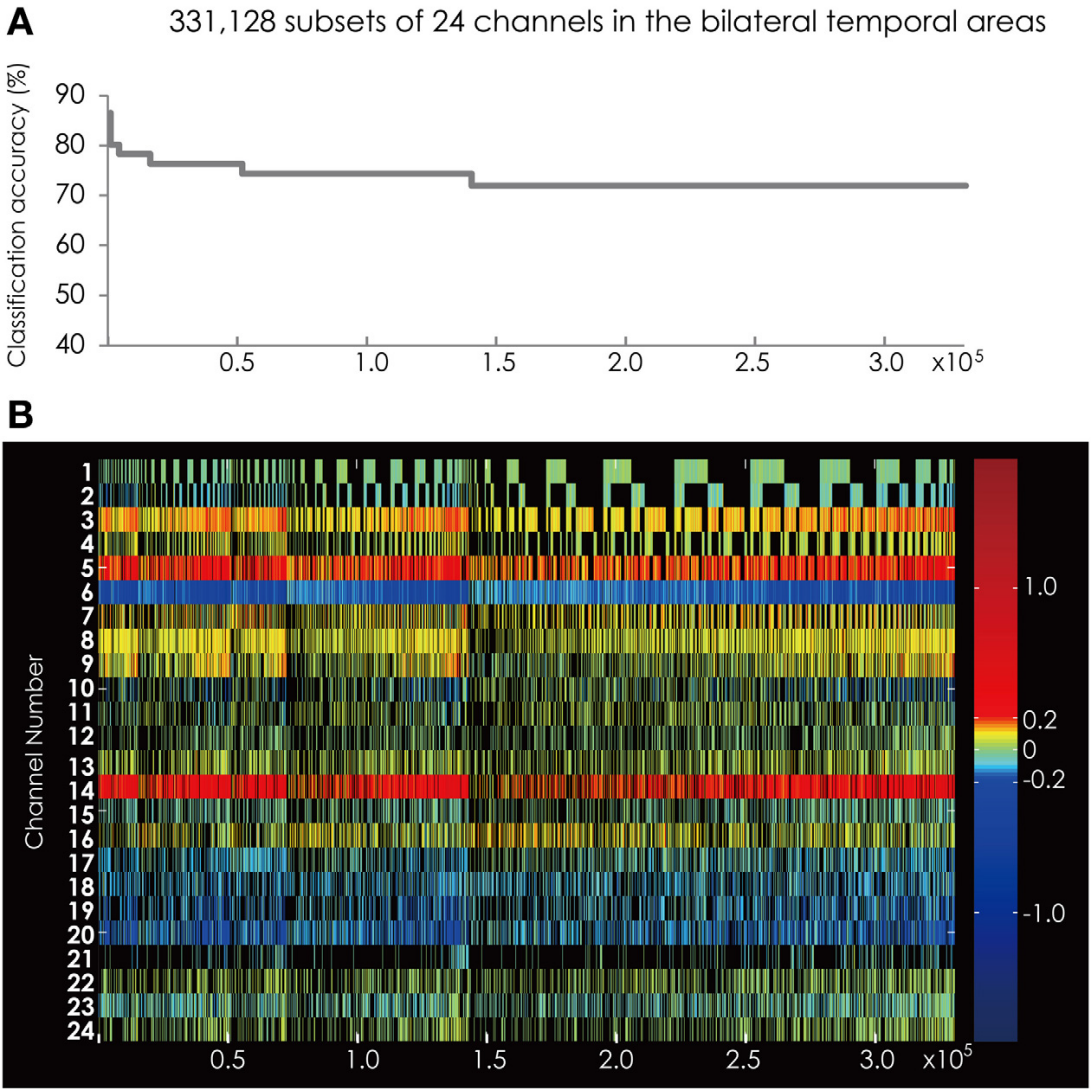
**(A)** The classification accuracy corresponding with 331,128 subsets consisted from the channels in the bilateral hemisphere. **(B)** The channel numbers in the subsets represented in **(A)**.

We found two subsets for classifying the data more correctly into two groups among the 16,777,215 subsets. The measurement channels that comprised those subsets listed in Table 1. These subsets had the best classification accuracy of 84%. Figure 6A shows the classification accuracy of the subsets with accuracy above 70%. The classification accuracy was higher when using the data set from the bilateral temporal areas than when using data sets from the one-side temporal area. This result indicated that the bilateral temporal areas interacted with each other and that the combined data analysis increased the amount of information about the classification.

Figure 6B shows the feature subsets corresponding to the classification accuracy represented in Figure 6A. As in the analysis on the data sets from each hemisphere, Ch. 6 appeared constantly and was colored blue.

**Figure 7:**
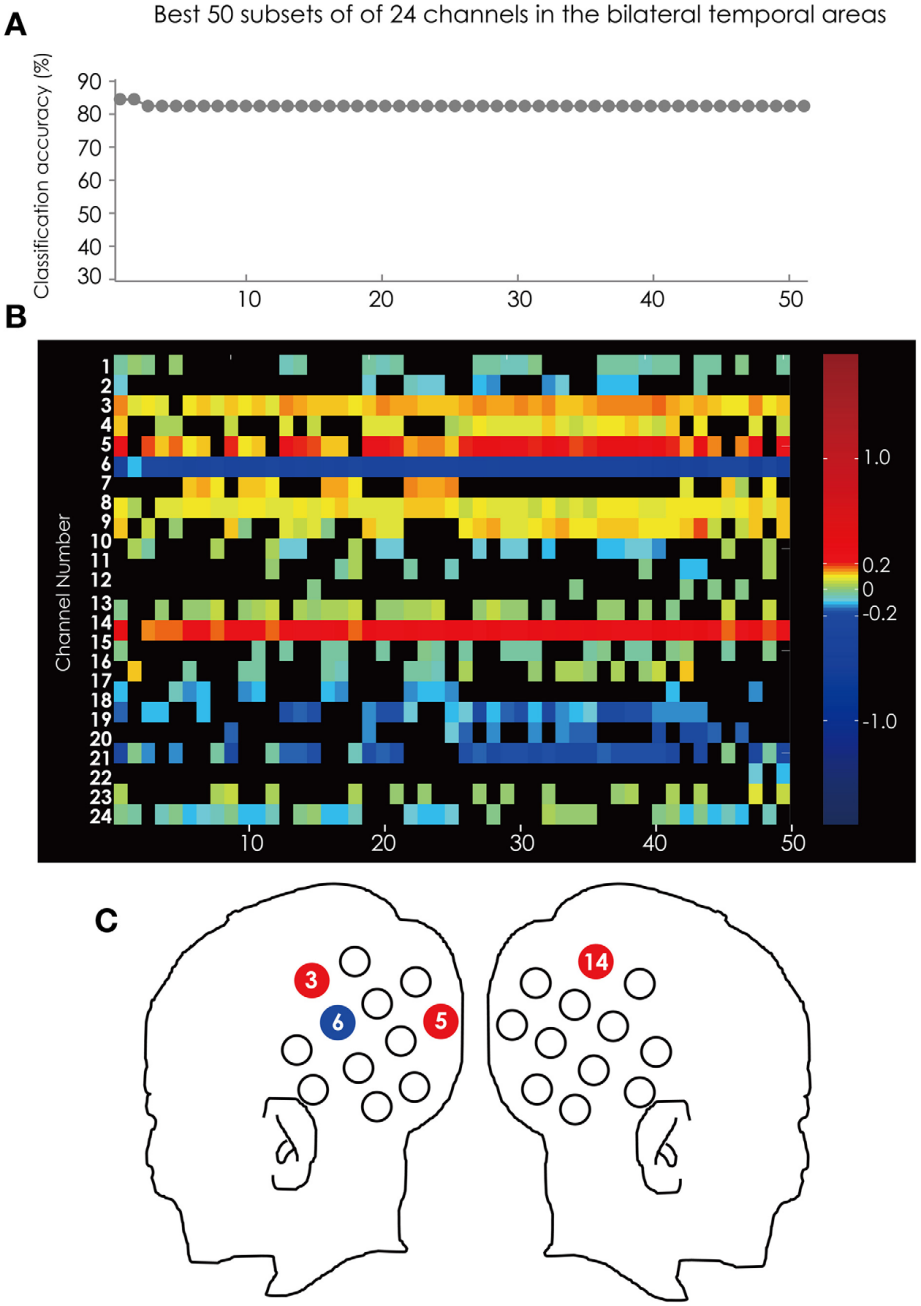
**(A)** The classification accuracy corresponding with the best 50 subsets consisted from the channels in the bilateral hemisphere. **(B)** The channel numbers in the subsets represented in **(A)**. **(C)** The position of the channels referred to in the text.

Figure 7 shows the classification accuracy for all 4095 subsets (Figure 7A) and the corresponding feature subsets (Figure 7B). Ch. 6 was in all of the 50 subsets and was most often used in the best 50 subsets. Ch. 3 and 14 were in 49 subsets. The position of these channels is illustrated in Figure 7C.

Ch. 3, 5, and 14 are red and only Ch. 6 is blue. This tendency is consistent through the top 50 subsets. This result indicated that the greater hemodynamic response in Ch. 3, 5, and 14 more often occurred in ADHD participants than in ASD participants, and that those at Ch. 6 are more often found in ASD participants than in ADHD participants.

### Lasso and sparse logistic regression

To demonstrate the effectiveness of the exhaustive search, we compared the exhaustive search with two existing methods, least absolute shrinkage and selection operator (LASSO, Tibshirani, 1996) and SLR (Yamashita et al., 2008). These are classification methods that incorporate feature selection as a part of the process of classifier training. We applied LASSO and SLR to datasets (1)–(3), and evaluated classification accuracies by using 5-fold CV.

By using LASSO, the classification accuracies were (1)57.5 and (2)66%. These accuracies rank (1)1106th (in the top 27.0%) and (2)147th (in the top 3.59%) of the 4095 accuracies obtained by the exhaustive search. The classification accuracy in dataset (3) was 70%, and this ranks 139,815th (in the top 0.833%) of the 16 million accuracies obtained by the exhaustive search.

By using SLR, the classification accuracies were (1)52 and (2)64%. These accuracies rank (1)1978th (in the top 48.3%) and (2)372th (in the top 9.08%) of the 4095 accuracies obtained by the exhaustive search. The classification accuracy in dataset (3) was 66%, and this ranks 698,955th (in the top 4.17%) of the 16 million accuracies obtained by the exhaustive search.

These results showed that, in datasets (2) and (3), the classification accuracies using LASSO and SLR were high and were located near the top of all the accuracies obtained by the exhaustive search. In comparison, in dataset (1) the classification accuracies were low compared to those in datasets (2) and (3), and were in the middle of all the accuracies obtained by the exhaustive search. These findings highlighted the fact that by using LASSO and SLR, the classification accuracy is not always high compared to the best result obtained by the exhaustive search, depending on the dataset. This fact was not revealed unless performing the exhaustive search. This means that neither LASSO nor SLR could uncover the latent structures relevant to discrimination between ASD and ADHD in the high-dimensional fNIRS data.

### Channel-wise analysis of 24 channels

With the aim of comparing the effectiveness of SVM with a standard method, we also performed a channel-wise *t*-test on each channel. A two-tailed two-sample *t*-test was conducted for the difference of Z-scores between the ADHD participants and ASD participants during the 3–10 s of the test trials. For all 24 channels, a *t*-test was performed. To reduce the risk of a Type I error, we performed the corrections using the false discovery rate (FDR) (Singh and Dan, 2006).

We did not find any significant difference in the mean Z-scores between ADHD participants and ASD participants. To compare the hemodynamic change with the baseline activation, we conducted a two-tailed one-sample *t*-test against the baseline. The *t*-test was repeated for each of 24 channels by applying the FDR procedure (Singh and Dan, 2006). However, we did not find any significant hemodynamic change from the baseline either in ADHD participants or ASD participants.

## Discussion

In this study, we applied SVM to real hemodynamic data obtained in Shimamura et al. (2012, under review) and tried to classify the data into two groups: ADHD participants and ASD participants. We exhaustively searched the optimal subset of measurement channels and evaluated each classification accuracy using 5-fold CV. We showed that the classification accuracy when using the best subset of channels was 84%, while that using all 24 channels was 62%. Additionally, we applied two feature selection methods, LASSO (Tibshirani, 1996) and SLR (Yamashita et al., 2008), which are intended to be applied to high-dimensional data, and confirmed that the best subset of channels was not selected when using these methods. Furthermore, the channel-wise analysis did not find any significant channels that showed distinctive activation for groups.

In the right hemisphere, SVM classification indicated that the greater weight value was consistently in three channels: Ch. 15, 16, and 18. These channels contribute to the classification in opposite directions because of their opposite signs of weight vectors in constricting the decision boundary. The sign of weight vector corresponding to Ch. 15 and 16 were positive, while that corresponding to Ch. 18 was negative. This result indicates that hemodynamic data obtained from Ch. 15 and 16 might increase for ADHD children and those obtained from Ch. 18 might decrease. To classify the input data into an ADHD group or an ASD group, we can focus on only the activity of these three channels, rather than all the measurement channels. (Using only these channels, we can classify the hemodynamic data into an ADHD group and an ASD group with 68% classification accuracy.) Ch. 15 and 16 correspond to the temporal-parietal junction (TPJ). The TPJ is the area responsive to theory of mind (ToM) (Saxe and Kanwisher, 2003) and is involved in familiar face recognition (Gobbini et al., 2004; Gobbini and Haxby, 2007). Although the neural response to a maternal face has not been investigated with school-aged children, previous study with adults (Ramasubbu et al., 2007) demonstrated that a maternal face evoked a stronger and broader hemodynamic activation than did unfamiliar faces. On the other hand, Bartels and Zeki (2004) demonstrated that maternal attachment and romantic love commonly activated the brain’s reward system, yet deactivated regions associated with negative emotions, social judgment, and ToM. Based on these previous studies, we can assume that increased hemodynamic response in the TPJ of ADHD participants might be related to the atypical attachment of ADHD children to their mother (Shimamura et al., 2012).

In the left hemisphere, though no channels appeared consistently through the best 50 subsets, some channels appeared alternately. In the best 17 subsets Ch. 3, 5, 7 appeared, while in the 18th to 28th subsets only Ch. 5 appeared dominantly. Below the 29th subsets, Ch. 6 appeared again and various unstable channels appeared alternately. These channels behave like small “patches.” These small-scale activities by some patches reflect the sparse expression of the information in/from the left hemisphere (Kitazono, 2013).

The imbalance of channels that contributes to the classification between the hemispheres might reflect the differential stage of face processing. Previous studies demonstrated that the left hemisphere predominates when faces are processed featurally, whereas the right hemisphere predominates when faces are processes configurally (Koenig and Hillger, 1991; Rossion et al., 2000; Scott and Nelson, 2006). Based on these findings, we can suppose that the greater number of channels in the left hemisphere might input raw (lower level) information and process featural properties such as eyes and mouth independently, whereas the lesser number of channels in the right hemisphere might input the pre-processed information and process the configuration of faces.

The best classification accuracy was obtained by the SVM classification on all 24 channels. The classification accuracy was 84%. We had 50 data, and 42 out of those 50 were correctly classified into the diagnosis group to which the participants belonged. Let us compare with the results of the SVM classification in each hemisphere and in the bilateral hemisphere. Three channels (Ch.15, 16, and 18) are important in the SVM classification in the right hemisphere, while four channels (Ch. 3, 5, 6, and 7) are important in the left hemisphere. Also, four channels (Ch. 3, 5, 6, and 14) are important in the SVM classification in the bilateral hemisphere. Only three (Ch. 3, 5, 6) out of the seven channels (Ch. 3, 5, 6, 7, 15, 16, 18) are common between the one-sided hemisphere and the bilateral hemisphere analysis. Moreover, one channel (Ch. 14) is not important in the right hemisphere analysis. We found that the discrepancies in the results of the SVM classification in the bilateral hemisphere that the channels used in the optimal subset were not consistent with those used in the optimal subset in each hemisphere. It would be logical that different input results in different output (Kitazono, 2013).

In this study, we aimed to classify the hemodynamic data into two distinct participant groups: ADHD participants and ASD participants. We exhaustively searched the optimal subset of fNIRS measurement channel and evaluated the classification accuracy using 5-fold CV. We successfully found the optimal subset for the classification of the real hemodynamic data with 84% accuracy. We can conclude that SVM and exhaustive search provides an effective method for hemodynamic data classification obtained from multichannel NIRS measurement.

### Conflict of interest statement

The authors declare that the research was conducted in the absence of any commercial or financial relationships that could be construed as a potential conflict of interest.
